# Food Nutrients and Bioactive Compounds for Managing Metabolic Dysfunction-Associated Steatotic Liver Disease: A Comprehensive Review

**DOI:** 10.3390/nu17132211

**Published:** 2025-07-03

**Authors:** Erdenetsogt Dungubat, Kohei Fujikura, Masahiko Kuroda, Toshio Fukusato, Yoshihisa Takahashi

**Affiliations:** 1 Department of Molecular Pathology, Tokyo Medical University, Shinjuku-ku, Tokyo 160-8402, Japan; dungubat.erdenetsogt.3p@tokyo-med.ac.jp (E.D.); kuroda@tokyo-med.ac.jp (M.K.); 2 General Medical Education and Research Center, Teikyo University, Itabashi-ku, Tokyo 173-8605, Japan; fukusato@med.teikyo-u.ac.jp

**Keywords:** metabolic dysfunction-associated steatotic liver disease, metabolic dysfunction-associated steatohepatitis, nutrition, fatty acid, polyphenol, vitamin, zinc, liver fibrosis, dietary intervention

## Abstract

Metabolic dysfunction-associated steatotic liver disease (MASLD) and its progressive form, metabolic dysfunction-associated steatohepatitis (MASH), are growing global health concerns. However, pharmacological therapies for MASLD/MASH have not yet been established. Dietary interventions and their bioactive components have been explored as strategies to mitigate MASLD and MASH progression. Although specific nutrients and bioactive compounds have exhibited potential therapeutic benefits, they also exacerbate adverse outcomes. In this comprehensive review, we synthesize the protective and exacerbating or sometimes dual effects of key macronutrients, including fatty acids (saturated, unsaturated, and trans fats) and carbohydrates (fructose, glucose, and sucrose), and bioactive compounds and micronutrients, in the context of MASLD management. The evidence suggests that coffee-derived compounds, such as caffeine and chlorogenic acid, may attenuate liver injury. However, the effects on MASLD severity are inconsistent. Diets high in saturated fatty acids exacerbate MASLD pathogenesis, whereas moderate intake (7–10% of total energy) may confer metabolic benefits. Other bioactive compounds and micronutrients have been explored for their diverse roles in hepatic lipid metabolism and insulin sensitivity. Although current evidence supports the therapeutic potential of specific dietary nutrients and bioactive compounds in the management of MASLD, inconsistencies in results highlight the need for more robust, well-controlled studies, including clinical trials, to clarify the preventive and therapeutic standards for balanced food interventions in MASLD management. In particular, well-designed clinical trials are necessary before clinical application. Although this is a narrative review and the literature retrieval may be biased, we covered a wide variety of substances.

## 1. Introduction

In 2023, the terms metabolic dysfunction-associated steatotic liver disease (MASLD) and its advanced form, metabolic dysfunction-associated steatohepatitis (MASH), were introduced to replace nonalcoholic fatty liver disease and nonalcoholic steatohepatitis. This revision underscores the growing recognition of metabolic dysfunction as a central driver of these conditions. Several academic societies have recently published clinical practice guidelines for the management of MASLD [1,2].

Changes in dietary patterns in recent decades, notably the increased consumption of ultra-processed foods, high-fructose diets, and energy-dense meals, combined with sedentary lifestyles, have substantially contributed to the increased prevalence of MASLD and MASH worldwide. Currently, MASLD affects approximately 38% of the global adult population and approximately 7–14% of children and adolescents. By 2040, the number of adults with MASLD is expected to increase to over 55%, highlighting an increasingly important public health concern [3]. In a large-scale cross-sectional ecological study, Younossi et al. found a strong correlation between MASLD prevalence and indicators of food insecurity, particularly the reliance on ultra-processed foods in high socio-demographic index countries [4]. These observations underscore the pivotal role of dietary quality in modulating the risk and severity of MASLD.

The pathogenesis of MASLD and MASH is complex and multi-factorial, involving metabolic dysregulation, gut–liver axis dysfunction, and immune-mediated mechanisms; however, our understanding of these processes is incomplete. The only recommended treatment approaches are lifestyle modifications, particularly dietary changes and increased physical activity. This highlights the urgent need to develop nutritional strategies for modulating key metabolic and inflammatory pathways involved in the pathophysiology of MASLD. In this context, research has increasingly focused on dietary interventions and bioactive food components as potential strategies for mitigating MASLD and MASH progression. Specifically, the effects of specific nutrients, functional foods, and dietary supplements on disease development have been explored.

Ranneh et al. reviewed the therapeutic potential of polyphenols in MASLD [5], and Mullin et al. reviewed the mechanistic roles of dietary fatty acids and sugars in MASLD progression [6]. These reviews provide valuable insights into the beneficial effects of specific dietary components. Most studies and reviews have emphasized the beneficial effects of specific nutrients and bioactive compounds in the management of MASLD. However, authors are increasingly reporting conflicting or even adverse effects. These inconsistencies underscore the complex and context-dependent nature of nutrient–liver interactions and highlight the need for more comprehensive, balanced evaluations that consider both the protective and potentially harmful effects of dietary factors on MASLD pathophysiology.

In this comprehensive narrative review, we aim to synthesize the current scientific evidence on the complex and sometimes dual roles of key macronutrients (e.g., fatty acids and carbohydrates), bioactive compounds (e.g., caffeine, chlorogenic acid [CGA], ethanol, polyphenols, like curcumin, resveratrol, silymarin, and polyamines, like spermidine), and micronutrients (e.g., vitamins D, E, and C and the trace elements zinc and selenium) in the management of MASLD. By critically evaluating the available literature, we aimed to elucidate the mechanisms of action in hepatic lipid metabolism, insulin sensitivity, oxidative stress, inflammation, and fibrosis while also highlighting discrepancies and knowledge gaps. While several previous studies have discussed the role of different dietary nutrients in the management of MASLD [7,8,9], this review is unique as we emphasize preclinical studies with detailed mechanism descriptions. Furthermore, we describe the effects of ethanol and polyamines (including data from our studies), which have not been widely described in previous review articles. Therefore, in this paper, we focus on the most promising and well-studied nutrients, like vitamins and polyphenols, as well as nutrients that have been rarely covered in previous review articles. Our findings inform future research directions and help develop more precise, evidence-based nutritional recommendations for managing this prevalent and complex metabolic disorder.

## 2. Effects of Macronutrients on MASLD

The liver uses three major macronutrients for energy: carbohydrates, fats, and proteins. Macronutrient composition, specifically carbohydrates and fatty acids, significantly influences hepatic lipid metabolism, inflammation, and insulin sensitivity, thereby playing a crucial role in the development and progression of MASLD. Recent clinical and experimental studies have investigated the influence of specific macronutrients on MASLD outcomes, highlighting both beneficial and harmful effects. The underlying mechanisms and comparative findings from preclinical and clinical studies are summarized in Table 1.

### 2.1. Fatty Acids

Fatty acids are central to the pathogenesis and progression of MASLD and influence hepatic lipid metabolism, insulin sensitivity, oxidative stress, and inflammation. The type and quantity of consumed fatty acids can have diverse effects on liver health.

#### 2.1.1. Saturated Fatty Acids

High SFA intake, particularly palmitic acid (C16:0) and stearic acid (C18:0), is strongly implicated in MASLD progression. SFAs serve as substrates for DNL and contribute significantly to hepatic triglyceride accumulation [10]. In addition to simple accumulation, SFAs induce lipotoxicity through several mechanisms. They can activate pro-inflammatory pathways, notably TLR4 signaling, leading to the production of cytokines (e.g., TNF-α and IL-6) and immune-cell recruitment [11,12]. SFAs also promote endoplasmic reticulum stress and mitochondrial dysfunction, contributing to oxidative stress and hepatocyte apoptosis [13]. Although relevant data from humans are limited, Rosqvist et al. (2014) reported increased liver fat accumulation and visceral adiposity in humans who overate SFAs compared with those who consumed unsaturated fat [14]. Furthermore, SFAs can directly impair insulin signaling and exacerbate systemic insulin resistance [15]. Dietary guidelines generally recommend limiting SFA intake to <10% of the total energy intake for metabolic health [16], which is particularly beneficial for MASLD management. Extremely low-fat diets, including those very low in saturated fats, can also impair the absorption of fat-soluble vitamins (A, D, E, and K) and affect hormone production or lead to metabolic imbalances, thereby impacting overall health [17].

#### 2.1.2. Unsaturated Fatty Acids

**Figure 1:**
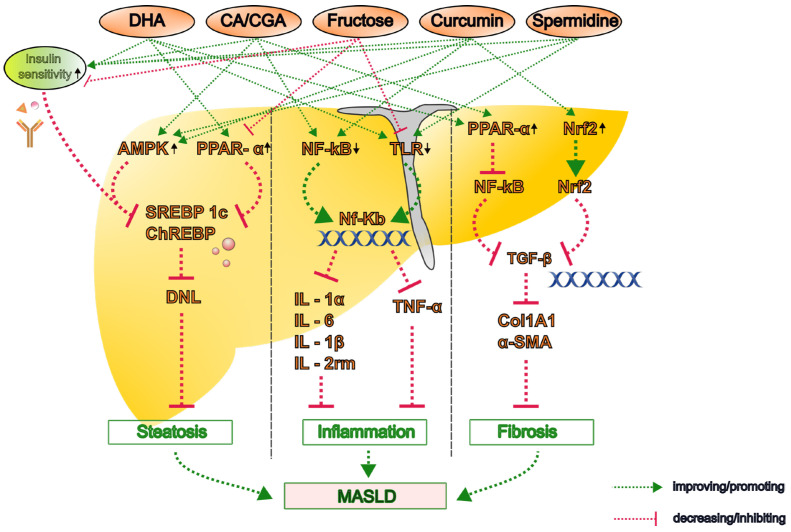
The proposed molecular mechanisms by which key dietary bioactive compounds influence the pathogenesis of metabolic dysfunction-associated steatotic liver disease (MASLD). Representative compounds—including caffeine, chlorogenic acid (CGA), polyphenols (curcumin, resveratrol, and silymarin), omega-3 polyunsaturated fatty acids (PUFAs), and polyamines (spermidine and putrescine)—modulate lipid metabolism, oxidative stress, inflammation, and fibrogenesis through multiple pathways. Upward arrow denotes an increase or upregulation and the downward arrow indicates decrease or downregulation in the activity or expression. α-SMA, alpha-smooth muscle actin; AMPK, AMP-activated protein kinase; CA, caffeine; CGA, chlorogenic acid; ChREBP, carbohydrate-responsive element-binding protein; Col1A1, collagen type I alpha 1; DHA, docosahexaenoic acid; DNL, de novo lipogenesis; IL, interleukin; NF-κB, nuclear factor kappa B; Nrf2, nuclear factor erythroid 2-related factor 2; PPAR, peroxisome proliferator-activated receptor; SREBP-1c, sterol regulatory element-binding protein 1c; TGF, transforming growth factor; TLR, toll-like receptor; TNF, tumor necrosis factor.

Unsaturated fatty acids, including monounsaturated fatty acids (MUFAs) and polyunsaturated fatty acids (PUFAs), generally exhibit more favorable metabolic profiles than saturated fats. MUFAs, predominantly oleic acid found in olive oil, are a key component of the Mediterranean diet and have been associated with reduced MASLD severity [18]. Mechanistically, MUFAs may improve insulin sensitivity and lipid profiles by displacing saturated fatty acids (SFAs) in cellular membranes and influencing signaling pathways [19,20]. PUFAs are categorized into omega-6 (n-6), primarily linoleic acid, and omega-3 (n-3), including eicosapentaenoic acid (EPA) and docosahexaenoic acid (DHA). Although n-6 PUFAs can produce pro-inflammatory eicosanoid compounds, n-3 PUFAs generally exert anti-inflammatory effects [21]. Patients with MASH have lower hepatic levels of long-chain n-3 PUFAs than those with simple steatosis [22]. PUFAs, particularly EPA and DHA, have therapeutic potential. They activate peroxisome proliferator-activated receptor alpha (PPARα) to enhance fatty acid oxidation, suppress sterol regulatory element-binding protein 1c (SREBP-1c) to reduce de novo lipogenesis (DNL), and activate G protein-coupled receptor 120 (GPR120) to mediate anti-inflammatory effects [23,24] (Figure 1). Most clinical trials have suggested that n-3 PUFA supplementation in both children and adults with MASLD/MASH can ameliorate the clinical and pathological findings of MASLD, but some studies showed no significant effects, suggesting limited and mixed evidence [25,26,27,28,29]. These clinical trials have some additional limitations. For example, in the clinical trial by Nobili et al., there was no control group (placebo group) and only 20 subjects [28]. In a clinical trial of omega-3-rich *Camelina sativa* by Musazadeh et al., the observational period was only 12 weeks [26]. Even in double-blind, placebo-controlled trials, the risk of bias cannot be denied because the matching of factors, such as age, sex, comorbidities, and lifestyle, may be insufficient.

A recent experimental study demonstrated that n-3 PUFAs attenuate steatosis in MASH model mice fed a high-fat diet (HFD) by stimulating the proliferation and differentiation of pre-adipocytes, thereby promoting the recruitment of new adipocytes and reducing hepatic lipid accumulation [30]. Furthermore, hepatic inflammation was notably attenuated by DHA rather than EPA in the atherogenic HFD- and Western diet-induced MASH model mice. This is because DHA lowers hepatic inflammatory markers, including interleukin (IL)-1α, IL-1β, IL-2rm, monocyte chemoattractant protein (MCP)-1, tumor necrosis factor (TNF)-α, CD68, and osteopontin [31,32]. Furthermore, in Ldlr−/− mice fed a Western diet, DHA suppressed the hepatic expression of CD14 (both messenger RNA (mRNA) and protein) and the toll-like receptors (TLR)2 and TLR4 and reduced the nuclear abundance of nuclear factor kappa B (NF-κB)-p50 and its precursor mRNA [32,33] (Figure 1).

DHA, but not EPA, has been shown to attenuate Western diet-induced hepatic fibrosis, as evidenced by the decreased gene and protein expression of collagen type I alpha 1 chain (Col1A1) and improved histological outcomes [32,34]. The antifibrotic effect of DHA is associated with its ability to attenuate hepatic transforming growth factor (TGF)-β signaling [33]. Furthermore, a recent preclinical study demonstrated that DHA-mediated inhibition of betacellulin disrupts the integrin pathway in macrophages and impairs TGF-β-driven collagen production by hepatic stellate cells, which synergistically contributes to the development of liver fibrosis [35] (Figure 1).

Experimental studies consistently highlight DHA as a more potent therapeutic n-3 PUFA than EPA for mitigating hepatic steatosis, inflammation, and fibrosis in MASLD. Although preclinical models have provided strong evidence for DHA’s hepato-protective properties, further clinical studies are required to validate these findings and establish the optimal intake parameters, including effective dosing and appropriate n-6/n-3 PUFA ratios for managing MASLD.

#### 2.1.3. Trans Fatty Acids

Industrially produced trans fatty acids (iTFAs), which are formed during partial hydrogenation of vegetable oils, are particularly detrimental to metabolic health. Although regulatory actions have significantly reduced the iTFA content in many food supplies, the levels of residual and naturally occurring TFAs from ruminant sources remain unclear [36]. The consumption of iTFAs is associated with an increased risk of cardiovascular disease, insulin resistance, and inflammation [37]. In the context of MASLD, iTFAs exacerbate hepatic steatosis by increasing DNL, impairing fatty acid oxidation, and promoting inflammation [38]. Animal studies have shown that high-TFA diets induce steatohepatitis features [39]. Although the human data are limited, minimizing TFA intake is a crucial dietary recommendation for preventing and managing MASLD [40].

### 2.2. Carbohydrates

Carbohydrates, particularly simple sugars such as fructose, glucose, and sucrose, play pivotal roles in the development and progression of MASLD. Excessive intake of these sugars, especially high-fructose corn syrup and sweetened beverages, is closely linked to hepatic steatosis, insulin resistance, and systemic inflammation.

#### 2.2.1. Fructose

Saccharides and dietary fiber can be classified as simple or complex carbohydrates. For example, fructose is a monosaccharide classified as a simple carbohydrate found in honey, fruits, and root vegetables. The carbohydrate load describes the storage of exogenous saccharides and their influence. Although fructose has a low glycemic index, excessive consumption, primarily from sugar-sweetened beverages and processed foods containing high-fructose corn syrup, is a primary dietary driver of MASLD [41]. Unlike glucose metabolism, fructose metabolism largely bypasses the primary regulatory step of glycolysis (phosphofructokinase-1), leading to a rapid influx of DNL substrates [42]. Fructose activates the key lipogenic transcription factors SREBP-1c and carbohydrate-responsive element-binding protein (ChREBP) [41,43] (Figure 1). Fructose metabolism also generates uric acid, which induces mitochondrial oxidative stress [44]. Furthermore, fructose consumption contributes to insulin resistance, alters gut microbiota composition, increases gut permeability and endotoxemia, and promotes hepatic inflammation [45,46]. According to a meta-analysis, high fructose intake is significantly associated with increased liver fat and insulin resistance [47].

Experimental studies have demonstrated that chronic fructose intake significantly influences the expression of numerous genes involved in key metabolic pathways, including glycolysis, lipogenesis, β-oxidation/lipolysis, fructolysis, and gluconeogenesis. These effects appear to be modulated by factors such as nutritional status and sex [48,49,50]. High-fructose diets are also strongly associated with MASH development because they promote hepatic DNL via SREBP1c and ChREBP [51,52,53,54] (Figure 1). Fructose-induced alterations in hepatic metabolism contribute to the development of insulin resistance and gut microbiota dysbiosis [55]. As fructose metabolism predominantly occurs in the liver, it bypasses key regulatory steps in glycolysis, resulting in unregulated lipid synthesis and hepatic lipid accumulation [56].

Chronic fructose consumption has been shown to induce oxidative stress and inflammatory responses in experimental models. In C57BL/6 mice, a high-fructose diet activated TLR4 signaling in the liver, leading to increased fibrogenesis, and the severity of MASH was exacerbated [56,57]. According to Takahashi et al., fructose-fed animal models reliably exhibit pronounced hepatic steatosis and increased hepatic expression of inflammatory cytokines, such as IL-6 and TNF-α [58]. Kawasaki et al. reported that rats fed a high-fructose diet developed significantly increased macrovesicular steatosis, hepatic triglyceride accumulation, and lobular inflammation. These animals also exhibited an increased liver-to-body weight ratio and higher prevalence of lipogranulomas, closely mirroring the histopathological features of MASH in humans [59]. These findings highlight the harmful effects of a high-fructose diet on liver pathology and disease progression (Figure 1).

Recent findings from an ongoing study by our group (Osaka Metropolitan University) suggest that a high-fructose diet and decreased male hormone levels following castration synergistically exacerbated hepatic steatosis in mice. This effect appears to be mediated, at least in part, by disruptions in gut microbiota homeostasis, emphasizing the intricate interplay among diet, hormonal regulation, and gut–liver axis disturbances in MASH progression. Future research should aim to elucidate the precise molecular mechanisms linking fructose metabolism, gut–liver axis dysfunction, and hormonal regulation to identify potential therapeutic strategies for mitigating MASLD/MASH progression.

#### 2.2.2. Glucose

Glucose is the primary circulating sugar, and its hepatic metabolism is closely regulated by insulin. Although glucose can contribute to DNL, especially under conditions of high carbohydrate intake and hyperinsulinemia, it is generally considered less lipogenic than fructose on a gram-for-gram basis [42]. Despite being less lipogenic, excessive glucose intake, particularly from refined sources, contributes to hyperinsulinemia and lipogenesis, indirectly worsening MASLD. High-glycemic-load diets promote hepatic insulin resistance and fat accumulation. Animal studies have shown that glucose-rich diets aggravate steatosis, particularly when combined with HFDs [60]. Takahashi et al. reported that rats fed a high-fructose/high-glucose diet developed histopathological features consistent with those of humans with MASH, including macrovesicular steatosis, perisinusoidal fibrosis, and lobular inflammation, particularly localized in zone 1 of the hepatic lobule. Notably, this high-fructose/high-glucose diet increased serum alanine aminotransferase (ALT) levels and the hepatic expression of pro-inflammatory cytokines, such as IL-6, despite the animals exhibiting lower body weights than starch-fed controls. This effect was accompanied by increased epididymal adipose tissue weight, mimicking visceral obesity in metabolic syndrome [61]. Diets rich in carbohydrates with a high glycemic index, which causes rapid spikes in blood glucose and insulin levels, can promote DNL and contribute to hepatic steatosis and insulin resistance over time [62]. Considering the overall carbohydrate intake and favoring low-glycemic index sources is crucial for managing MASLD.

#### 2.2.3. Sucrose

Sucrose (table sugar) is a disaccharide consisting of one glucose molecule and one fructose molecule. Upon ingestion, sucrose is rapidly hydrolyzed in the intestine. Consequently, high sucrose intake leads to the same detrimental metabolic effects associated with high glucose and particularly high fructose consumption, including potent stimulation of DNL and contribution to insulin resistance and hepatic fat accumulation [41,42]. Chronic sucrose intake promotes hepatic steatosis and inflammation comparable to high-fructose diets [63]. The additive effects of glucose and fructose accelerate oxidative stress and fibrotic signaling [63,64]. Limiting the intake of added sugars, including sucrose, is a key dietary strategy for managing MASLD.

## 3. Effects of Food Bioactive Compounds and Micronutrients on MASLD

In addition to macronutrients, various bioactive compounds and micronutrients are being investigated. Bioactive compounds derived from plant-based foods have shown considerable therapeutic potential in modulating the pathogenesis of MASLD by targeting key pathological mechanisms, including oxidative stress, inflammation, metabolic dysregulation, and fibrosis. Micronutrients, including essential vitamins and trace elements, modulate the pathophysiology of MASLD by regulating oxidative stress responses, enhancing insulin sensitivity, modulating lipid metabolism, and attenuating hepatic inflammation and fibrosis.

### 3.1. Bioactive Ingredients in Beverages

Bioactive compounds found in common beverages, such as coffee, tea, and plant-based drinks, have garnered significant interest for their potential roles in modulating the pathogenesis of MASLD.

#### 3.1.1. Caffeine and Chlorogenic Acid

**Figure 2:**
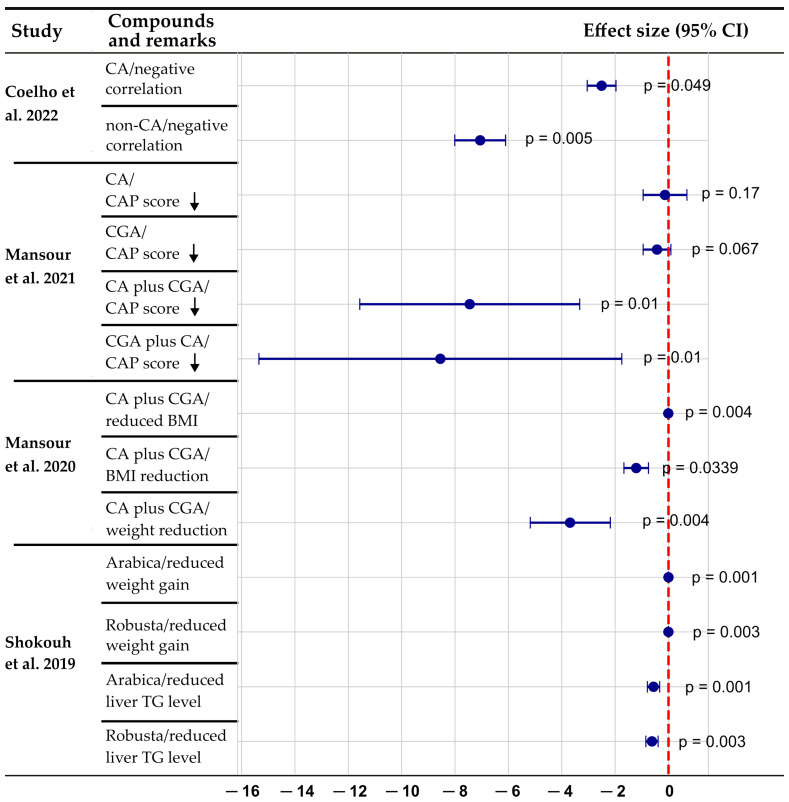
A forest plot of the association between coffee components and MASLD parameters. Negative values indicate the beneficial effects of coffee/caffeine and chlorogenic acid on MASLD parameters. The combination of caffeine plus chlorogenic acid appears to have stronger effects than either component alone. Both robusta and arabica coffee varieties show significant benefits for liver triglyceride levels and weight reduction. *p*-values displayed on the right side of each data point indicate statistical significance of the observed effects, with values < 0.05 considered statistically significant. Data compiled from Coelho et al. (2022) [69], who reported negative correlations between fatty liver index and caffeine metabolites (*p* = 0.049, *p* = 0.005); Mansour et al. (2021) [70], who demonstrated effects of caffeine and chlorogenic acid on controlled attenuation parameter scores (p values ranging from 0.01 to 0.17); Mansour et al. (2020) [71], who showed significant BMI and weight reduction with caffeine plus chlorogenic acid supplementation (*p* = 0.004, *p* = 0.0339); and Shokouh et al. (2019) [72], who documented reduced liver triglyceride levels and weight gain with both arabica and robusta coffee varieties (*p* values ranging from 0.001 to 0.003). Downward arrow indicates a decrease of CAP score. BMI, body mass index; CA, caffeine; CAP, controlled attenuation parameter; CGA, chlorogenic acid; TG, triglyceride.

Caffeine and CGA are major bioactive compounds found in coffee, tea, and certain plant-based foods that have gained increasing attention, given their potential hepatoprotective effects in MASLD. Several epidemiological studies have suggested that habitual coffee consumption is inversely correlated with the progression of MASH, including reduced risk of fibrosis and hepatocellular carcinoma [65,66,67,68]. These beneficial effects appear to be mediated by both independent and combined actions of caffeine and CGA, which exert regulatory effects on lipid metabolism, oxidative stress, inflammation, and fibrogenic pathways. However, clinical and observational studies have primarily focused on whole coffee consumption, and experimental studies are required to elucidate the precise molecular mechanisms through which these compounds exert their hepatoprotective effects. A growing body of clinical and epidemiological evidence suggests that caffeine and CGA intake are associated with improvements in hepatic steatosis, inflammation, and metabolic health. These benefits are reflected in reductions in the controlled attenuation parameter and improvements in key clinical indicators, such as serum triglyceride levels and body mass index (Figure 2).

Most experimental studies on caffeine and CGA have reported inhibitory effects on MASLD/MASH [73,74,75,76,77,78,79]. Several animal studies have demonstrated that caffeine intake improves hepatic lipid metabolism by reducing DNL and enhancing fatty acid β-oxidation [73,74,76,77]. However, several studies have reported exacerbating effects [77,80,81,82,83,84,85].

Xin et al. showed that in an MASH mouse (C57BL/6J) model fed a high-fat high-carbohydrate diet, caffeine significantly reduced hepatic triglyceride accumulation by suppressing SREBP-1c and ChREBP, key regulators of lipogenesis, while upregulating PPARα and carnitine palmitoyl transferase (CPT)1, which promote lipid oxidation [76]. Velázquez et al. reported similar findings in rats fed an HFD plus 10% liquid fructose, showing that caffeine activated AMP-activated protein kinase (AMPK) signaling, leading to reduced hepatic lipid accumulation and improved insulin sensitivity [77] (Figure 1).

According to other animal studies, CGA counteracts hepatic steatosis by suppressing lipogenesis and promoting β-oxidation [75,78]. Xu et al. showed that CGA administration in HFD-fed obese mice significantly reduced hepatic triglyceride content by activating AMPK signaling and suppressing SREBP-1c and the liver X receptor (LXR)α [78].

The molecular mechanisms and pathways through which caffeine and CGA exert their antioxidant, anti-inflammatory, and antifibrotic effects have been detailed in previous reviews [86,87]. These mechanisms include the modulation of oxidative stress, the suppression of inflammation via crosstalk between IL-6 production in muscle and liver signal transducer and activator of transcription (STAT)3 activation, and the inhibition of fibrogenic pathways.

In a combined in vivo and in vitro study, Yang et al. demonstrated that CGA attenuates liver fibrosis by inhibiting the microRNA (miR)-21-regulated TGF-β1/mothers against the decapentaplegic homolog (Smad)7 signaling pathway. This inhibition resulted in the downregulation of alpha-smooth muscle actin (α-SMA) and collagen I expression in liver tissue, thereby mitigating fibrotic progression [79] (Figure 1).

Recent systematic reviews, including the one by Salvoza et al., have emphasized evidence of the effects of caffeine and CGA on MASH [80]. This review analyzed five experimental studies on the effects of caffeine and CGA; however, two of these studies reported no significant effect of caffeine or CGA administration in reducing the risk of progression to more severe stages of MASLD or MASH [77,85].

Our group previously reported that caffeine and CGA exacerbated liver inflammation and fibrosis in mice fed a choline-deficient, L-amino acid-defined HFD (CDAHFD). Both caffeine and CGA showed exacerbating effects attributed to the absence of significant changes in the expression of key lipid metabolism-related genes, including *Cpt1a*, *Srebp-1c*, and *fatty acid synthase* (*Fasn*) mRNA across experimental groups. Moreover, caffeine and CGA supplementation significantly worsened markers of liver cell injury and inflammation, as evidenced by elevated serum ALT levels and an increased number of CD45R-positive cells in the liver. IL-6 may be associated with exacerbated effects because hepatic expression levels of *IL-6* mRNA were significantly higher in the caffeine group than in the CDAHFD group. However, the association was not definitive since *IL-6* mRNA expression levels in the caffeine group were almost the same as in the control diet group [81].

Hu et al. reported that prenatal caffeine exposure in rats increased the susceptibility of female offspring to MASLD through activation of the glucocorticoid receptor-CCAAT/enhancer binding protein-sirtuin 1 (GR-C/EBP-SIRT1) pathway [82]. However, conflicting findings have been reported in human studies. For instance, in a study involving individuals who were overweight or obese with type 2 diabetes, although caffeine metabolites were inversely correlated with the fatty liver index, direct coffee consumption was not significantly associated with MASLD parameters, including fibrosis or steatosis severity [83].

Mubarak et al. reported that CGA supplementation did not confer protective effects against metabolic syndrome features in obese mice fed an HFD. Notably, mice receiving CGA supplementation alongside the HFD exhibited increased hepatic lipid content and worsened steatosis compared to those on the HFD alone, suggesting impaired fatty acid oxidation [84].

The heterogeneity of the study conditions (e.g., experimental models, dosages, duration of treatment, and methodological approaches) likely contributed to the discrepancies observed in the research investigating the effects of caffeine and CGA on MASLD and MASH. Further investigations are required to clarify the distinct and combined effects of caffeine and CGA administration on MASH pathogenesis and progression across different species, age groups, and sexes.

#### 3.1.2. Ethanol

Although MASLD diagnosis requires the exclusion of considerable alcohol consumption, the interaction between metabolic risk factors and alcohol intake is being increasingly recognized. Modest alcohol consumption in individuals with MASLD may accelerate disease progression compared to abstaining from alcohol [88]. This combined pathological effect is thought to involve multiple mechanisms, including enhanced oxidative stress, dysregulated lipid metabolism, and immune activation [89]. Alcohol metabolism generates reactive oxygen species and acetaldehyde; increases the nicotinamide adenine dinucleotide (NADH/NAD+) ratio, favoring steatosis; impairs mitochondrial function; increases gut permeability, leading to elevated endotoxin (lipopolysaccharide) levels; and sensitizes the liver to inflammatory stimuli [90,91].

Although MASLD/MASH is distinct from alcoholic liver disease, the emerging evidence has suggested that ethanol consumption may have paradoxical effects on disease progression. However, clinical and epidemiological studies have reported conflicting outcomes, meaning that the effect of alcohol intake on MASLD and MASH remains highly controversial. For example, studies have reported that low doses of alcohol may have protective effects, but others have demonstrated the exacerbation of liver injury at higher levels of consumption [88,92,93,94,95,96,97,98,99,100,101]. Although excessive alcohol consumption is a well-established driver of hepatic inflammation, fibrosis, and disease progression, some epidemiological studies have suggested that low-to-moderate alcohol intake, particularly red wine, may exert hepatoprotective effects [94,95,96,97,98]. However, other studies have contradicted these findings, indicating that even light-to-moderate alcohol intake may exacerbate fibrosis and hepatic injury [88,99,100,101]. Light alcohol consumption was generally defined as alcohol intake of <20 g/day, and abstinence was defined as no alcohol intake during a study.

A recent meta-analysis of cohort studies reported that moderate alcohol consumption increased the risk of advanced fibrosis (pooled odds ratio 1.56; 95% confidence interval 1.08–2.26) and hepatocellular carcinoma (hazard ratio 1.39; 95% confidence interval 1.22–1.57) in patients with MASLD. However, this association was not observed in cross-sectional studies. Notably, although alcohol use was linked to a higher risk of cirrhosis and hepatocellular carcinoma, it appeared to lower the risk of steatohepatitis [92]. Given the inconsistencies in human studies, future studies should aim to construct experimental models to delineate the direct effects of ethanol on MASH pathophysiology.

Several experimental studies have investigated the effects of ethanol in conjunction with HFDs [102,103,104]. The first investigation of the effects of low-dose ethanol on MASH was conducted by our group using an HFD-fed db/db mouse model. The findings demonstrated that short-term (8-week) light ethanol administration (a high-fat liquid diet supplemented with 0.1% ethanol ad libitum) significantly improved MASH parameters, including reductions in serum aspartate aminotransferase (AST) and ALT levels, and hepatic inflammation. The number of CD45R- and myeloperoxidase-positive inflammatory cells in both the hepatic lobule and portal tract was markedly lower in the low-ethanol group than in the HFD group [105].

Daniels et al. examined binge ethanol consumption in multiple MASH model mice fed an HFD, a high-fat diet with cholesterol, and a high-fat/cholesterol diet with trans fats. Their results revealed that ethanol exacerbated the inflammatory and fibrogenic properties of an atherogenic diet-induced MASH model, suggesting that alcohol acts synergistically with metabolic stressors to accelerate hepatic injury [106]. Yeh et al. demonstrated that a single ethanol binge (5 g/kg body weight) induced severe liver injury, evidenced by marked elevations in AST and ALT, in C57BL/6J mice fed a Western diet for 3 weeks [107]. However, these studies primarily examined high ethanol doses (15–31.25% ethanol), limiting their relevance to the potential short-term effects of low or moderate alcohol consumption.

Although light-to-moderate ethanol intake may exert hepatoprotective effects, the long-term effects remain uncertain. Researchers should aim to develop well-characterized MASH animal models to investigate the effects of low levels of alcohol consumption over the long and short term to determine whether it is beneficial or contributes to disease progression. Abstinence from alcohol is generally recommended for patients with MASLD [108], and the World Health Organization’s global status report on alcohol and health in 2018 specifically indicates that no amount of alcohol is safe [109].

#### 3.1.3. Tea and Its Ingredients

Green tea is a popular beverage worldwide, especially in Asia. Green tea contains various bioactive compounds, including polyphenols. Among them, catechins (a kind of polyphenol) are known for their health-promoting effects. Tea catechins include epicatechin, epigallocatechin, epicatechin gallate, and epigallocatechin gallate (EGCG). Of these, EGCG, in particular, is highly bioactive and accounts for approximately 50% of the total polyphenols in tea leaves [110].

Green tea extract was found to prevent MASLD in mice, and the preventive effects were attributed to the activation of mitochondrial respiratory chain complexes, SIRT1, and AMPK [111,112,113]. EGCG improved MASLD/MASH in mice and rats, and the effects were associated with lower concentrations of profibrogenic, oxidative, and pro-inflammatory mediators and a decreased absorption of bile acids and lipids [114,115,116].

Several clinical trials have shown the effects of tea and its ingredients on MASLD/MASH. For example, a double-blind, placebo-controlled, randomized clinical trial found that green tea extract (GTE) supplementation (500 mg GTE tablet per day) significantly reduced ALT and AST levels after a 12-week period, whereas the placebo did not [117]. However, this study had limitations because the number of patients was relatively small (80 patients), and the observational period was short. In a double-blind, randomized, controlled clinical trial, sour tea supplementation (450 mg capsule per day) significantly decreased serum triglyceride, ALT, and AST levels compared with the placebo [118]. However, this study also had limitations due to the small number of patients (70 patients) and short observational period (8 weeks). In a double-blind placebo-controlled study, the consumption of 700 mL of green tea with high-density catechins (1080 mg catechins) per day significantly decreased body fat and serum ALT levels and significantly improved the liver-to-spleen computed tomography attenuation ratio compared with the placebo and green tea with low-density catechins (200 mg catechins) [119]. However, clinical data on the effects of the ingredients of tea on MASLD are scarce, and further studies are required.

### 3.2. Polyphenols

Polyphenols are a diverse group of plant-derived compounds widely recognized for their potent antioxidant, anti-inflammatory, and metabolic regulatory properties. Notably, curcumin, resveratrol, and silymarin have been extensively studied in preclinical and clinical settings. These representative polyphenols have demonstrated potential in preventing hepatic steatosis and attenuating disease progression, making them attractive candidates for adjunctive dietary or therapeutic interventions in MASLD. A summary of their reported effects is presented in Table 2.

#### 3.2.1. Curcumin

Curcumin, the principal curcuminoid derived from turmeric (*Curcuma longa*), has garnered attention for its hepatoprotective properties in MASLD. Preclinical studies have demonstrated that curcumin can attenuate MASLD/MASH by modulating lipid metabolism, reducing oxidative stress, and suppressing inflammatory pathways. Specifically, curcumin inhibits the nucleotide-binding domain, leucine-rich-containing family, and pyrin domain-containing-3 (NLRP3) inflammasome and activates AMPK and nuclear factor erythroid 2-related factor 2 (Nrf2) pathways, leading to decreased lipid accumulation and inflammation in hepatic tissues [120] (Figure 1).

A meta-analysis of 52 preclinical studies involving 792 animals found that curcumin effectively improved key pathological markers associated with MASH, including hepatic steatosis, inflammation, and fibrosis [121]. In one of the reviewed studies, curcumin supplementation significantly attenuated the effects of HFDs on glucose disposal, body weight/fat gain, and insulin resistance in C57BL/6J mice over 28 weeks. The study also found that curcumin reduced intrahepatic lipid content and downregulated mRNA levels of ChREBP and SREBP-1c, two key transcription factors involved in hepatic lipogenesis, as well as liver pyruvate kinase, a downstream target of ChREBP [122].

Curcumin was also found to exhibit potent anti-inflammatory properties in MASH rat models by suppressing key pro-inflammatory cytokines, such as TNF-α, IL-6, and IL-1β. Curcumin reduced oxidative stress by enhancing antioxidant defenses, including increased glutathione levels and decreased malondialdehyde levels, a marker of lipid peroxidation. Curcumin enhanced the efficacy of oxidative stress factors, including superoxide dismutase, sirtuin 2 (Sir2), glutathione peroxidase, and Nrf2 [123] (Figure 1). Elkordy et al. reported that curcumin administration alleviates MASH by modulating endoplasmic reticulum stress, oxidative stress, apoptosis, and autophagy. These effects are achieved through the downregulation of protein kinase RNA-like endoplasmic reticulum kinase (PERK) and C/EBP homologous protein (CHOP) expression, the restoration of Beclin-1 levels, and the improvement of the ratio between Bcl-2-associated X protein (Bax)/B-cell lymphoma 2 protein (Bcl-2) in HFD-induced MASH model mice [124].

Curcumin has also been shown to suppress α-SMA and collagen I protein expression levels in cardiac fibroblasts treated with TGF-β1, indicating that it inhibits fibrogenesis by targeting the TGF-β/Smad3 pathway [125]. Another study found that the hepatoprotective effects of pomegranate extract and curcumin against thioacetamide-induced liver fibrosis are linked to their ability to modulate the Nrf2/heme oxygenase-1 (HO-1), NF-κB, and TGF-β/Smad3 signaling pathways [126]. Collectively, these studies indicate that curcumin attenuates fibrosis by modulating the TGF-β/Smad3 signaling pathway, leading to a significant reduction in the expression of the fibrogenesis markers α-SMA and collagen I.

Numerous clinical and epidemiological studies have investigated whether curcumin can mitigate MASLD and MASH [127,128,129]. For instance, a randomized, placebo-controlled trial involving 50 patients with MASLD reported that 12 weeks of curcumin supplementation (1500 mg/day) significantly reduced hepatic steatosis, serum liver enzymes, and inflammatory markers compared with a placebo [130]. Another study using a bioavailable form of curcumin, Meriva^®^, at a dose equivalent to 50 mg/day of pure curcumin for 8 weeks improved serum metabolite profiles associated with MASLD [131]. The results of human trials have indicated that curcumin supplementation may improve serum liver function markers, serum lipid profiles, and markers of steatosis [131].

Despite these promising findings, inconsistent outcomes regarding curcumin efficacy have also been reported. For example, although curcumin significantly reduced serum AST levels (mean difference: −3.36 U/L, 95% confidence interval: −5.35 to −1.36, *p* = 0.001), the magnitude of this effect varied across studies, and the overall heterogeneity was considerable [132]. The inconsistencies in the results may be due to challenges associated with curcumin’s low bioavailability or variability in study design. Regardless, further large-scale, controlled trials are required to establish standardized dosing regimens and confirm the therapeutic efficacy of curcumin in MASLD management.

#### 3.2.2. Resveratrol

Resveratrol, a natural polyphenol found in grapes, berries, and peanuts, has been extensively studied for its potential benefits in metabolic disorders, including MASLD [133]. Preclinical studies have shown that resveratrol can improve insulin sensitivity, reduce hepatic lipid accumulation, and exert anti-inflammatory effects by modulating pathways such as SIRT1 and AMPK. SIRT1 activation can improve insulin sensitivity, enhance mitochondrial biogenesis and function, promote fatty acid oxidation, and reduce DNL [134] (Figure 1).

However, the clinical evidence remains inconclusive. Several randomized controlled trials have yielded mixed results regarding the efficacy of resveratrol in improving liver function and reducing hepatic steatosis in patients with MASLD [134,135]. Factors contributing to these inconsistencies include variations in study populations, dosages, treatment durations, and the low bioavailability of the compound [136]. To address these challenges, research has focused on enhancing the bioavailability of resveratrol through novel nano-drug delivery systems [137], which may improve its therapeutic potential in MASLD. Nonetheless, further well-designed clinical trials are required to validate these approaches and determine the role of resveratrol in MASLD treatment.

#### 3.2.3. Silymarin

Silymarin, a flavonolignan complex extracted from milk thistle (*Silybum marianum*), has been traditionally used for its hepatoprotective effects. In the context of MASLD, silymarin exhibits antioxidant, anti-inflammatory, and antifibrotic properties. A recent preclinical study revealed that silymarin, administered at a dose of 80 mg/kg, was more effective at mitigating liver steatosis and reducing lipid accumulation than a lower dose of 30 mg/kg silymarin or a combination of silybin and isosilybin A [138]. Notably, silymarin was shown to regulate bile acid metabolism by reducing the concentration of 7-ketodeoxycholic acid (7-KDCA), thereby modulating farnesoid X receptor (FXR) activity through a negative feedback mechanism [138].

Clinical studies have suggested that silymarin can modulate the gut microbiota composition and reduce liver stiffness, potentially through interactions with the FXR pathway [139,140]. However, a meta-analysis of clinical trials found that silymarin may improve liver enzymes (e.g., ALT and AST) in patients with MASLD, but its effects on liver histology (steatosis, inflammation, and fibrosis) are less consistent [141,142]. A randomized, double-blind, placebo-controlled trial demonstrated that silymarin supplementation significantly decreased liver stiffness in patients with MASLD, indicating potential benefits in reducing hepatic fibrosis [140]. However, another study found that essential phospholipids were more effective at improving liver function and reducing fibrosis than silymarin when assessed using FibroScan^®^ [143]. These discrepancies highlight the need for further research to determine optimal dosing strategies and treatment durations and to elucidate the mechanisms underlying the effects of silymarin on MASLD.

#### 3.2.4. Hesperidin

The polyphenol hesperidin is a flavanone glycoside found in citrus fruits that consists of aglycon hesperetin and disaccharide rutinose. Hesperidin possesses antioxidant and anti-inflammatory properties and is expected to have beneficial effects on various diseases [144,145].

Preclinical studies showed that hesperidin prevents MASLD in experimental animals. This was attributed to its inhibition of pyroptosis and endoplasmic reticulum stress-induced inflammation and the promotion of fatty acid β-oxidation via the activation of SIRT1/peroxisome proliferator-activated receptor gamma coactivator 1-alpha (PGC1α) [146,147,148]. A recent study showed that hesperetin also prevents MASLD in rats [149].

In a randomized, placebo-controlled, double-blind clinical trial, MASLD patients administered a 1 g hesperidin capsule for 12 weeks showed significant improvement in ALT, γ-glutamyltransferase, total cholesterol, triglyceride, high-sensitivity C-reactive protein, TNF-α, NF-κB, and hepatic steatosis compared with the placebo group [150]. However, the study has limitations because the number of patients was small (50 patients), and the period of observation was short. A randomized controlled clinical trial with one hundred MASLD patients, which was performed by the same group and used 1 g hesperidin supplementation for 12 weeks, found a significant reduction in body mass index, glucose homeostasis parameters, and hepatic steatosis compared with the control group [151]. Although promising effects were gained in these clinical studies, studies with more patients and a longer observational period are required in the future. Histopathological evaluations via liver biopsy are also desirable.

### 3.3. Polyamines

Polyamines, such as putrescine, spermidine, and spermine, are small aliphatic amines naturally present in endogenous metabolism and various dietary sources, including whole grains, soy products, mushrooms, and fermented foods. Polyamines have garnered growing interest in the context of MASLD owing to their capacity to influence hepatocellular lipid accumulation, mitochondrial function, and fibrotic progression. In particular, experimental studies have suggested that polyamines confer protective effects against hepatic steatosis and liver injury, highlighting their potential as novel nutritional or pharmacological targets for managing MASLD. A summary of their reported effects is presented in Table 3.

#### 3.3.1. Putrescine

Putrescine, the simplest polyamine, is derived from ornithine decarboxylation and serves as a precursor for spermidine and spermine biosynthesis. Clinical studies directly assessing the effects of putrescine supplementation in patients with MASLD are lacking. However, studies using animal models have revealed that the oral administration of putrescine improves gut mucosal morphology by suppressing inflammatory responses [152,153] and augmenting albumin synthesis and mitochondrial DNA copy number in the liver [154]. Our research group recently investigated the role of putrescine supplementation in a CDAHFD-induced db/db mouse model of MASH (unpublished data). Our findings demonstrated that putrescine significantly reduced the markers of inflammation, hepatocellular injury, and fibrosis. Histological evaluation revealed a significant reduction in hepatocyte ballooning, accompanied by a reduction in steatosis grade, intralobular inflammation, perisinusoidal fibrosis, and fibrosis stage in the putrescine-treated group compared with those in the CDAHFD group. The protective effects of putrescine in MASH appear to be partially mediated through the PPAR-α and PPAR-γ signaling pathways.

A recent experimental study by Dasdelen et al. demonstrated that putrescine supplementation reduced liver damage by exerting anti-inflammatory and antioxidant effects, including NF-κB and IL-6 inhibition [155]. However, another study reported that elevated endogenous hepatic putrescine and increased ornithine decarboxylase levels correlate with greater MASLD severity [156]. Future research should focus on elucidating the regulatory pathways governing putrescine metabolism, optimizing therapeutic dosing strategies, and clarifying the long-term impact of putrescine on liver health.

#### 3.3.2. Spermidine

Spermidine, a naturally occurring polyamine derived from putrescine, plays a pivotal role in cellular homeostasis, autophagy, and mitochondrial functions. Spermidine levels decline with age, and spermidine supplementation has protective effects against various age-related diseases. A key mechanism is the induction of autophagy, including lipophagy, which helps clear lipid droplets and damaged organelles [157]. Preclinical studies have indicated that spermidine can ameliorate hepatic steatosis and inflammation in MASLD models, potentially via autophagy induction and AMPK activation [158,159] (Figure 1). In dietary supplementation models, spermidine has been shown to enhance autophagic flux, reduce hepatic steatosis, and suppress the expression of pro-inflammatory cytokines, such as TNF-α and IL-1β, thereby mitigating liver injury and fibrosis [159] (Figure 1). Moreover, spermidine’s ability to modulate lipid metabolism through SIRT1 activation and the mammalian target of rapamycin inhibition is associated with improved hepatic insulin sensitivity and reduced oxidative stress.

Although these findings collectively highlight the promising therapeutic potential of spermidine, especially as a modulator of autophagy and inflammation, robust clinical trials are lacking. Such trials will be required to elucidate the long-term safety profile and optimal dosing and identify interactions with polyamine metabolic pathways. A deeper understanding of these mechanisms is critical for translating preclinical success into viable dietary interventions for managing MASLD.

#### 3.3.3. Spermine

Spermine, a naturally occurring polyamine synthesized from spermidine, is involved in cellular homeostasis, including the regulation of oxidative stress, apoptosis, and immune responses. Preclinical studies have demonstrated that spermine supplementation can alleviate acute liver injury by inhibiting pro-inflammatory responses in liver-resident macrophages via ATG5-dependent autophagy pathways [160]. Spermine has also been shown to enhance liver barrier function, modulate amino acid transporters, and suppress apoptosis in animal models, suggesting hepatoprotective effects [161].

Kukoamines A and B are polyamine-conjugated alkaloids structurally linked to a spermine-like backbone. While spermine modulates immune cell polarization and enhances autophagy, kukoamines A and B reduce hepatic steatosis and oxidative stress by downregulating SREBP-1c and pro-inflammatory cytokines [162,163]. Despite these promising findings, direct clinical evidence of the efficacy of spermine in managing MASLD remains limited. Further research, including well-designed clinical trials, is required to elucidate the therapeutic potential and safety profile of spermine supplementation in patients with MASLD.

### 3.4. Vitamins

Vitamins play essential roles in maintaining hepatic function and systemic metabolic balance, and several vitamins have emerged as promising candidates for therapeutic intervention in MASLD. Vitamins D, E, and C have been extensively investigated for their potential to mitigate oxidative stress, inflammation, and fibrogenesis associated with steatosis and steatohepatitis. Relevant findings from the reviewed preclinical and clinical studies are summarized in Table 4.

#### 3.4.1. Vitamin D

Alongside its classical role in calcium homeostasis, vitamin D also influences insulin signaling, lipid metabolism, and immune modulation, with deficiencies frequently observed in patients with MASLD. Vitamin D deficiency, particularly low serum levels of 25-hydroxy vitamin D [25(OH)D] (optimal value: ≥30 ng/mL), is prevalent among individuals with MASLD and has been consistently associated with increased hepatic steatosis, insulin resistance, and inflammatory activity [164]. Several observational studies have reported that reduced 25(OH)D levels are correlated with greater severity of steatosis, inflammation, and fibrosis, further linking vitamin D status to the progression of liver disease and broader metabolic dysfunction [164,165,166,167]. Vitamin D receptor activation exerts anti-inflammatory effects, improves insulin sensitivity, and modulates the activation of hepatic stellate cells [168]. However, clinical trials of vitamin D supplementation in MASLD have produced inconsistent results with regard to improvements in liver histology and biochemical markers, suggesting a complex relationship influenced by factors such as dosage, baseline levels, and patient genetics [169]. Clinical trials have primarily examined the effects of vitamin D supplementation on the serum concentrations of inflammatory markers and lipid profiles in patients with MASLD [165,170,171]. In these clinical trials, subjects were orally administered 50,000 IU of vitamin D3 (cholecalciferol) weekly; 50,000 IU of vitamin D3 every 14 days; or 2000 of IU vitamin D3 daily. In a randomized controlled trial, Barchetta et al. found that oral vitamin D supplementation did not significantly improve hepatic steatosis or metabolic parameters in patients with MASLD or type 2 diabetes, indicating limited therapeutic efficacy in this subgroup [165].

Rodent studies have suggested that vitamin D deficiency or supplementation significantly influences MASH development and progression. Cordeiro et al. investigated the effects of vitamin D supplementation in Western diet-fed obese rats. Their findings revealed that vitamin D supplementation reduced visceral adiposity and normalized leptin and circulating TNF-α levels, suggesting a protective role against diet-induced obesity and inflammation [172]. Other experimental studies have indicated that vitamin D and its receptor play crucial roles in suppressing fibrogenic signaling, thereby attenuating hepatic fibrosis [173]. In addition, vitamin D regulates miRNAs involved in liver lipid metabolism and hepatocyte turnover, including miR-122, miR-34a, and miR-21 [174].

In conclusion, vitamin D supplementation appears to confer protective effects against metabolic disturbances associated with MASH. However, the clinical findings underscore the need for further well-designed clinical trials to clarify its role as a therapeutic agent for managing MASLD.

#### 3.4.2. Vitamin E

Vitamin E (α-tocopherol), a lipid-soluble antioxidant, improves liver histology in non-diabetic patients with MASH by reducing oxidative injury and inflammatory responses [175]. As a major lipophilic antioxidant, vitamin E protects cell membranes from lipid peroxidation because oxidative stress is a key driver of MASH progression [176]. The PIVENS trial demonstrated that, compared with a placebo, vitamin E (800 IU/day) improved liver histology (steatosis, inflammation, and ballooning) in non-diabetic adults with MASH [176]. Based on this study outcome, guidelines recommend vitamin E for this specific patient subgroup [177]. Vitamin E supplementation has also been associated with reductions in the serum markers of liver inflammation and improvements in liver histology in patients with MASLD [178]. However, concerns about potential long-term risks at high doses (e.g., increased all-cause mortality and hemorrhagic stroke risk) warrant caution and limit its widespread use [179].

#### 3.4.3. Vitamin C

Vitamin C (ascorbic acid), a water-soluble antioxidant, has garnered attention for its potential hepatoprotective effects in MASLD because of its capacity to neutralize reactive oxygen species and regenerate vitamin E. Given the prominent role of oxidative stress in MASLD pathogenesis, the antioxidant properties of vitamin C are of particular interest.

Preclinical studies have demonstrated that vitamin C supplementation mitigates hepatic steatosis and oxidative stress. For instance, in a choline-deficient diet-induced MASLD rat model, vitamin C administration markedly reduced the histological alterations associated with fatty liver disease. Moreover, the role of vitamin C in regenerating other antioxidants, such as vitamin E, indicates that it has potential synergistic effects in combating oxidative liver damage [180]. In addition, vitamins C and E can exert antioxidative effects at different sites because of their respective hydrophilic and lipophilic qualities [181].

Clinical investigations have yielded promising results. A randomized controlled trial involving patients with NAFLD reported that daily oral vitamin C supplementation improved liver health and associated metabolic parameters, including glucose metabolism and adiponectin levels [182]. A cross-sectional study also found that higher dietary vitamin C intake was inversely associated with the risk of MASLD, suggesting a protective role for vitamin C against liver fat accumulation [182,183].

Despite these encouraging findings, the evidence remains inconclusive regarding the efficacy of high-dose vitamin C supplementation as a standalone treatment for MASLD. Some studies have suggested that vitamin C is beneficial when combined with other antioxidants, such as vitamin E or N-acetylcysteine. However, dedicated trials are required to clarify its role and optimal dosage [110,184]. Furthermore, although observational studies have indicated a potential protective association between serum vitamin C levels and MASLD risk [183], a causal relationship has not yet been established. This should be the focus of future Mendelian randomization analyses.

In summary, vitamin C exhibits potential hepatoprotective properties in the context of MASLD through its antioxidant effects and its involvement in metabolic processes. However, further well-designed clinical trials are required to determine its therapeutic efficacy, optimal dosing strategies, and long-term safety for managing MASLD.

### 3.5. Essential Trace Elements

Deficiencies in essential trace elements involved in antioxidant defense and metabolic regulation have been implicated in MASLD. Among them, zinc and selenium have garnered considerable interest in MASLD research. They regulate oxidative stress, inflammation, and insulin signaling, which are key disease progression mechanisms. A summary of the relevant studies is presented in Table 4.

#### 3.5.1. Zinc

Zinc is a cofactor of numerous enzymes, including the antioxidant enzyme copper–zinc superoxide dismutase, and it plays a role in insulin signaling and immune function [185]. Zinc deficiency is common in patients with chronic liver disease and may exacerbate oxidative stress and insulin resistance in MASLD [186]. Some studies have suggested that zinc supplementation alone or combined with other antioxidants may improve liver enzymes and metabolic markers in patients with MASLD [187].

Zinc, an essential trace element, plays a critical role in various biological processes, including immune function, antioxidant defense, and lipid metabolism. The evidence from rodent studies indicates that zinc homeostasis significantly influences MASH development and progression. Preclinical research has suggested that zinc deficiency exacerbates hepatic steatosis, oxidative stress, and inflammation, which are key drivers of MASH pathology [188,189,190,191]. Zinc supplementation has potential hepatoprotective effects, including the regulation of lipid metabolism and the attenuation of inflammatory responses. The experimental models show conflicting results regarding the efficacy of zinc supplementation in mitigating MASH-related hepatic impairment. Although several studies have suggested beneficial effects on lipid and glucose metabolism, others have indicated limited or no impact on the metabolic dysregulation associated with HFD-induced liver disease [192].

Zinc can alleviate metabolic disturbances induced by an HFD, particularly lipid and glucose homeostasis. Qi et al. reported that dietary zinc supplementation in HFD-fed mice and zinc treatment in HepG2 cells effectively attenuated hepatic steatosis and improved glucose and lipid metabolism, suggesting a regulatory role for zinc in hepatic function and metabolic pathways [192]. Conversely, Bolatimi et al. found no significant differences in the expression levels of hepatic function and fatty acid transport/metabolism-related genes, such as the hepatocyte nuclear factor (Hnf)4α and PPARα, between the control and zinc-treated groups. The authors attributed these discrepancies to the shorter duration of zinc treatment (8 weeks) compared with longer-term supplementation studies, which may be necessary to observe significant metabolic improvement.

Inconsistent findings in experimental models suggest that the efficacy of zinc supplementation in MASH may depend on several factors, including treatment duration, dosage, dietary composition, and co-supplementation with other micronutrients. Further well-designed long-term studies are needed to clarify the precise role of zinc in metabolism and its therapeutic potential in managing MASH.

#### 3.5.2. Selenium

Selenium is an essential component of selenoproteins, including glutathione peroxidase, which are critical antioxidant enzymes [193]. Selenium deficiency can impair antioxidant defenses. Although some studies have linked low selenium status to increased MASLD risk or severity [193,194], other studies have reported conflicting findings or potential toxicity with high selenium intake [195]. Co-supplementation of zinc and selenium in high-calorie diet-fed rats reduced the serum levels of ALT and AST, fasting plasma glucose and insulin, homeostatic model assessment for insulin resistance (HOMA-IR), vascular endothelial growth factor, and malondialdehyde, further supporting the hepatoprotective effects of both the essential trace elements zinc and selenium [195,196]. However, more in-depth studies are required to fully understand the role of selenium supplementation in MASLD and optimize its use.

#### 3.5.3. Sodium

The association between dietary sodium and MASLD has garnered attention. According to a recent retrospective data search, subjects with MASLD exceeded the recommended daily allowance (RDA) for sodium (241% of RDA) [197]. A recent longitudinal cohort study also suggested that high sodium intake in patients with MASLD is associated with increased disease incidence but decreased all-cause mortality [198]. A recent bidirectional Mendelian randomization study revealed a significant association between “salt added to food” and increased MASLD risk (odds ratio: 1.5–2.1), and subsequently, “salt added to food” was considered a causal risk factor for MASLD [199]. Further experimental studies are required to evaluate the effects of dietary sodium on MASLD and identify the associated mechanisms.

## 4. Discussion and Conclusions

This paper comprehensively reviews the effects of various food nutrients and bioactive compounds against MASLD. However, as this was a narrative review, the method used for the literature retrieval was not systematic and may have been biased. Furthermore, while this review focuses on MASLD, it is important to note that an increasing body of research indicates that natural compounds, such as flavonoids, resveratrol, saponins, and β-carotene, exert protective effects against alcoholic liver disease (ALD), as well as MASLD, and could thus be used in targeted therapies [200]. This is probably because MASLD and ALD share numerous common mechanisms [201].

Natural compounds have limitations that must be considered: (1) The clinical efficacy of most natural compounds is limited due to their low bioavailability. (2) Defining the most appropriate drug form and dosage is difficult without standardized pharmaceutical technology. Thus, the optimal liver protection effect cannot be realized. (3) Most compounds have not been subjected to double-blind, placebo-controlled clinical trials to evaluate their efficacy, and the mechanisms of action of natural compounds are still unclear because studies are limited [202]. Consequently, there are safety concerns [203]. In addition, most peer-reviewed publications are biased toward positive results, and challenges are generally not discussed [204].

Oral administration is the most commonly used method for nutrients, but other methods must be considered, depending on the patient’s condition. Optimal dosing strategies, safety profiles, potential adverse effects, drug–nutrient interactions, and long-term safety should also be considered, as acceptable daily intake is set for many nutrients, and overdose may cause various toxic effects. For example, caffeine overdose can cause dizziness, increased heart rate, insomnia, and diarrhea. Caution is especially necessary in long-term administration.

MASLD and its more progressive form, MASH, represent a global health crisis. As pharmacological therapies for MASLD have not been established, attention has increasingly shifted to dietary interventions and nutritional strategies as viable therapeutic avenues. This comprehensive review underscores the multifaceted role of macronutrients, bioactive compounds, and micronutrients in mitigating or exacerbating MASLD pathogenesis and progression.

The primary causes of conflicting results in animal experiments are differences in animal models (e.g., species and strain, diet) and experimental methods (e.g., dosage, route of administration, and observational period). Researchers should use the most appropriate animal model for the study and the experimental method applicable to humans. The primary causes of conflicting results in clinical trials are the differences in the subjects (e.g., age, sex, comorbidities, and lifestyle) and study design (e.g., blinding, randomization, number of subjects, administration method, and dosage). A study design with minimal bias and the inclusion of many subjects is desirable. Researchers should specify the cause when conflicting results appear and determine the most reliable data.

The conclusions of this review are based primarily on preclinical studies. The gap between animal models and human disease is particularly relevant for MASLD research. Consequently, if researchers use animal models whose pathophysiology differs from human MASLD, translating the results to humans is difficult. Vacca et al. [205] generated an unbiased ranking of murine dietary models based on their proximity to MASLD and showed that Western diets align closely with human MASH. It is essential to use reliable animal models to translate the results of preclinical studies to humans.

The evidence consistently indicates that dietary fat, particularly the quality and type of fatty acids, significantly affects hepatic lipid metabolism and inflammation. PUFAs, especially DHA, have potent antisteatotic, anti-inflammatory, and antifibrotic properties in preclinical models, making them promising therapeutic candidates. However, further research is required to establish optimal intake parameters, including effective dosing and appropriate n-6/n-3 PUFA ratios, for managing MASLD. In contrast, excessive intake of SFAs and iTFAs exacerbates hepatic steatosis and inflammatory responses via mechanisms involving endoplasmic reticulum stress, TLR activation, and mitochondrial dysfunction. These findings support the current dietary guidelines advocating for reducing SFA and TFA intake in individuals with MASLD. However, studies have confirmed that extremely low-fat diets, including those very low in saturated fats, can impair the absorption of fat-soluble vitamins (A, D, E, and K) and affect hormone production or lead to metabolic imbalances.

Carbohydrate quality is another important factor to consider when managing MASLD. High fructose intake, particularly from sugar-sweetened beverages, is strongly associated with increased DNL, insulin resistance, and gut–liver axis disruption, thereby promoting MASH development. Although glucose and sucrose are less lipogenic than fructose alone, their combined metabolic burden contributes to hepatic fat accumulation and fibrosis. Therefore, limiting the consumption of high-glycemic foods and added sugars remains a crucial dietary goal.

In addition to macronutrients, numerous bioactive compounds exhibit context-dependent effects on liver pathology. Polyphenols such as curcumin, resveratrol, and silymarin generally exert hepatoprotective effects through anti-inflammatory, antioxidative, and antifibrotic mechanisms. However, the bioavailability limitations and conflicting clinical trial results warrant cautious interpretation and highlight the need for improved delivery systems and standardized protocols. Likewise, compounds such as caffeine and CGA exert protective and exacerbating effects, depending on the dosage, treatment duration, and experimental model. These inconsistencies highlight the necessity for rigorous and well-controlled investigations to delineate their exact role in MASLD therapy.

Polyamines, particularly putrescine, spermidine, and spermine, have emerged as novel dietary modulators of hepatic metabolism and immune regulation. Although animal studies have suggested beneficial roles in reducing hepatic inflammation and fibrosis, the duality of endogenous versus exogenous effects and the lack of clinical trials underscore the need for mechanistic clarity and translational research.

Micronutrients, including vitamins D, E, and C, and trace elements, such as zinc and selenium, also play critical roles in the pathophysiology of MASLD. Vitamin E is the supplement most widely shown to improve liver histology among non-diabetic patients with MASH. In preclinical studies, vitamin D and zinc show promising anti-inflammatory and antifibrotic effects, but the human data are inconsistent. Although essential, selenium has a narrow therapeutic window and exhibits potential effects when co-supplemented with zinc. Therefore, individualized assessments and supplementation strategies are recommended.

In summary, the current body of evidence supports the implementation of diets rich in unsaturated fatty acids, polyphenols, and essential micronutrients while optimizing harmful fats and simple sugars for managing MASLD. However, there are considerable gaps in the literature regarding the dosage, bioavailability, long-term safety, and efficacy of the different potential components of such diets in diverse populations. Future research should focus on well-designed clinical trials, standardized nutritional protocols, and integrative approaches considering genetic, environmental, and lifestyle factors to establish evidence-based dietary guidelines for MASLD prevention and treatment. In particular, DHA and vitamin E should be further investigated using well-designed clinical trials to bring them to clinical practice.

## Figures and Tables

**Table 1:** Summary of macronutrient intervention studies for MASLD.

Nutrients and Compounds	Intervention	Main Findings	Remarks	Ref.
SFA	High SFA intake	↑ Lipotoxicity, ↑ insulin resistance, ↑ endoplasmic reticulum stress	Very low (<7%) saturated fat levels can lead to metabolic imbalances	[10,11,12,13,14,15,16,17]
PUFAs (DHA, EPA)	Varied; DHA often more potent	↓ Steatosis, ↓ inflammation, ↓ fibrosis via PPARα, SREBP-1c inhibition, GPR120 activation	Dose/ratio uncertainties remain	[18,19,20,21,22,23,24,25,26,27,28,29,30,31,32,33,34,35]
Trans fatty acids	Varied; often industrial trans fatty acids	↑ DNL, ↑ inflammation, ↑ steatosis	—	[36,37,38,39,40]
Fructose	High intake, chronic	↑ DNL, ↑ inflammation, ↑ fibrosis, ↑ gut dysbiosis	—	[41,42,43,44,45,46,47,48,49,50,51,52,53,54,55,56,57,58,59]
Glucose	High-glucose/fructose diet	↑ inflammation, ↑ perisinusoidal fibrosis	—	[42,60,61,62]
Sucrose	Chronic high intake	↑ steatosis, ↑ oxidative stress, ↑ fibrosis	—	[41,42,63,64]

DHA, docosahexaenoic acid; DNL, de novo lipogenesis; EPA, eicosapentaenoic acid; GPR 120, G protein-coupled receptor 120; MASLD, metabolic dysfunction-associated steatotic liver disease; PPAR, peroxisome proliferator-activated receptor; PUFA, polyunsaturated fatty acid; SFA, saturated fatty acid; SREBP-1c, sterol regulatory element-binding protein 1c.

**Table 2:** Summary data from studies using polyphenol interventions for MASLD.

Nutrient and Compound	Intervention	Main Findings	Remarks	Ref.
Curcumin	1500 mg/day (clinical); varied preclinically	↓ Steatosis, ↓ inflammation, ↓ fibrosis via AMPK, Nrf2, TGF-β pathways	Bioavailability and dose variability	[120,121,122,123,124,125,126,127,128,129,130,131,132]
Resveratrol	Varied; often limited by bioavailability	↑ SIRT1, ↑ AMPK, ↓ lipids, ↓ inflammation	Mixed clinical results	[133,134,135,136,137]
Silymarin	30–80 mg/kg (preclinical)	↓ Lipids, ↓ 7-KDCA, modulates FXR	Mixed histological outcomes	[138,139,140,141,142,143]
Hesperidin	Varied; often 500–1000 mg/day for 12 weeks	Improved metabolic parameters; reduced liver enzymes (ALT, AST); decreased inflammatory markers	Limited clinical studies	[144,145,146,147,148,149,150,151]

ALT, alanine aminotransferase; AMPK, AMP-activated protein kinase; AST, aspartate aminotransferase; FXR, farnesoid X receptor; 7-KDCA, 7-ketodeoxycholic acid; MASLD, metabolic dysfunction-associated steatotic liver disease; Nrf2, nuclear factor erythroid 2-related factor 2; SIRT1, sirtuin 1; TGF, transforming growth factor.

**Table 3:** Summary data from polypamine intervention studies for MASLD.

Nutrient and Compound	Intervention	Main Findings	Remarks	Ref.
Putrescine	80 mg/kg in mice	↓ Inflammation, ↓ fibrosis via PPARα/γ, NF-κB inhibition	Endogenous levels may correlate with severity	[152,153,154,155,156]
Spermidine	Dietary supplementation (preclinical)	↑ Autophagy, ↓ inflammation, ↑ SIRT1/AMPK	Lack of clinical trials	[157,158,159]
Spermine	Supplemented or endogenous	↓ Apoptosis, ↑ autophagy, ↓ inflammation (via ATG5)	Limited direct MASLD evidence	[160,161,162,163]

AMPK, AMP-activated protein kinase; MASLD, metabolic dysfunction-associated steatotic liver disease; NF-κB, nuclear factor kappa B; PPAR, peroxisome proliferator-activated receptor; SIRT1, sirtuin 1.

**Table 4:** Summary data of the reviewed studies on vitamin and essential trace element interventions for MASLD.

Nutrients and Compounds	Intervention	Main Findings	Remarks	Ref.
Vitamin D	Various oral regimens	↓ Inflammation, ↑ insulin sensitivity, modulates vitamin D receptor pathways	Inconsistent clinical outcomes	[164,165,166,167,168,169,170,171,172,173,174]
Vitamin E	800 IU/day (PIVENS)	↓ Oxidative stress, ↓ inflammation, ↓ ballooning	Potential risk at high doses	[175,176,177,178,179]
Vitamin C	Supplement or dietary	↓ Reactive oxygen species, regenerates vitamin E, synergistic antioxidant	Limited as monotherapy	[180,181,182,183,184]
Zinc	Dietary supplement (varied)	↓ Inflammation, ↑ metabolism, antioxidant	Results depend on duration and diet	[185,186,187,188,189,190,191,192]
Selenium	Dietary/co-supplemented	↑ Glutathione peroxidase activity, ↓ oxidative stress	Narrow therapeutic window	[193,194,195,196]
Sodium	Dietary intake long-term observational survey	Higher or suboptimal intake of magnesium, potassium, and calcium was common in MASLD patients, highlighting targets for dietary intervention	Limited experimental observation	[197,198,199]

PIVENS, pioglitazone, vitamin E, or placebo for nonalcoholic steatohepatitis; MASLD, metabolic dysfunction-associated steatotic liver disease.

## Data Availability

All referenced articles are publicly available on PubMed (https://pubmed.ncbi.nlm.nih.gov) (accessed on 30 June 2025).

## Abbreviations

α-SMA, alpha-smooth muscle actin; ALD, alcoholic liver disease; ALT, alanine aminotransferase; AMPK, AMP-activated protein kinase; AST, aspartate aminotransferase; Bax, Bcl-2-associated X protein; Bcl-2, B-cell lymphoma 2 protein; CDAHFD, choline-deficient, L-amino acid-defined, high-fat diet; C/EBP, CCAAT-enhancer-binding protein; CGA, chlorogenic acid; CHOP, C/EBP homologous protein; ChREBP, carbohydrate-responsive element-binding protein; Col1A1, collagen type I alpha 1; CPT, carnitine palmitoyl transferase; DHA, docosahexaenoic acid; DNL, de novo lipogenesis; EGCG, epigallocatechin gallate; EPA, eicosapentaenoic acid; Fasn, fatty acid synthase; FXR, farnesoid X receptor; GPR 120, G protein-coupled receptor 120; GR, glucocorticoid receptor; GTE, green tea extract; HFD, high-fat diet; Hnf, hepatocyte nuclear factor; HO-1, heme oxygenase-1; HOMA-IR, homeostatic model assessment for insulin resistance; IL, interleukin; iTFA, industrially produced trans fatty acid; 7-KDCA, 7-ketodeoxycholic acid; LXR, liver X receptor; MASH, metabolic dysfunction-associated steatohepatitis; MASLD, metabolic dysfunction-associated steatotic liver disease; MCP, monocyte chemoattractant protein; miR, microRNA; MUFA, monounsaturated fatty acid; NADH/NAD+, nicotinamide adenine dinucleotide; NF-κB, nuclear factor kappa B; NLRP3, nucleotide-binding domain, leucine-rich-containing family, pyrin domain-containing-3; Nrf2, nuclear factor erythroid 2-related factor 2; 25(OH)D, 25-hydroxy vitamin D; PERK, protein kinase RNA-like endoplasmic reticulum kinase; PGC1α, peroxisome proliferator-activated receptor gamma coactivator 1-alpha; PPAR, peroxisome proliferator-activated receptor; PUFA, polyunsaturated fatty acid; RDA, recommended daily allowance; SFA, saturated fatty acid; Sir2, sirtuin 2; SIRT1, sirtuin 1; Smad, mothers against the decapentaplegic homolog; SREBP-1c, sterol regulatory element-binding protein 1c; TGF, transforming growth factor; TLR, toll-like receptor; TNF, tumor necrosis factor.
